# Supplementary material for: Are carbon emissions trading and green financial instruments synergistic? -Comprehensive quantitative research based on content analysis
Source: PLoS One. 2024 Mar 7;19(3):e0298601. doi: 10.1371/journal.pone.0298601 (PMC10919601; doi:10.1371/journal.pone.0298601)
Supplement: S1 Data — (ZIP) [file pone.0298601.s001.zip › Data/地级市Z_F工作报告.zip!/┴╔─■/┼╠╜⌡╩╨/└┤╫╘╣1⁄2╓┌║┼í╛┬φ┐╦╩2╛▌═°í┐_2017.docx]

各位代表：

现在，我代表市政府向大会报告工作，请予审议，并请市政协委员和其他列席人员提出意见。

一、2016年工作回顾

过去的一年，面对异常严峻复杂的经济形势，在省委、省政府和市委的正确领导下，我们认真学习贯彻习近平总书记系列重要讲话精神，牢固树立五大发展理念，全面落实“四个着力”和供给侧结构性改革要求，以13个专项行动为抓手，统筹做好稳增长、促改革、调结构、惠民生、防风险各项工作，实现了“十三五”平稳开局。

预计实现地区生产总值1100亿元；固定资产投资601亿元；一般公共预算收入100.5亿元；社会消费品零售总额375亿元；城镇常住居民人均可支配收入34738元；农村常住居民人均可支配收入14933元。各项主要经济指标总量和增速继续走在全省前列。我们的工作，得到了国务院和省委、省政府的高度评价，特别是在国务院第三次大督查中，盘锦成为全省唯一受到国务院通报表扬的地级市。

（一）稳增长取得新成效

全力稳定企业运行。建立市县两级领导帮扶企业责任制，在降成本、去杠杆、开拓市场等方面加大协调服务力度，共减免涉企税费5.7亿元。 辽河油田保持稳产。华锦集团利润增长5.8倍，北燃公司加工进口原油700万吨、利润增长3.9倍，经济效益大幅提升。

全力扩大有效投资。实施全面推进项目建设专项行动，亿元以上项目新开工133项、续建282项、竣工142项。中国兵器集团精细化工及原料工程项目获得20亿元国家专项建设基金，忠旺铝型材等项目加快建设，万达广场等项目竣工运营，成为新的经济增长点。创新投融资方式，获批发行3支债券，总额度33.7亿元。落实PPP项目15个，吸纳社会资本56.8亿元，政府和社会资本合作取得显著成效。

全力抓好招商引资。全市签约注册项目259项。与央企诚通集团在金融、旅游、物流、纸制品4个领域重大合作项目取得重要突破。实际利用外资1.8亿美元。进一步加大出口基地建设力度，进出口总额增长2.1倍。

（二）辽东湾新区建设取得新进展

把新区确立为全市落实“四个着力”先行区，大力实施百项工程，开启了第二个十年建设发展的新篇章。

不断完善港口功能。盘锦港11个5万吨级以上泊位加快建设，10万吨级航道工程启动实施，吞吐能力达到5000万吨。一类口岸一次性通过国家验收。保税物流中心封关运行。

加快壮大产业规模。和运新材料、长春石化、瑞德化工等龙头企业效益稳步提升，台湾联成化学、中储粮综合产业基地、北大荒粮食仓储物流基地等72个项目进展顺利。平台经济、口岸经济、研发经济快速发展，石化产品交易平台实现交易额103亿元。

实施打造辽东湾创业新城专项行动。新区体制机制不断完善，行政审批和公共服务更加便捷高效。基础设施、商贸旅游设施等建设全面推进，曙光科技创业区等创新创业平台不断壮大，新区吸引力和综合承载力进一步增强。

（三）重点领域改革实现新突破

实施促进投资与服务便利化专项行动。优化政府部门机构设置，取消和调整116项市级行政职权事项，取消审批前置要件33项。在全省率先实行全程代办+容缺审批+企业承诺制度，组建市、县(区)两级行政审批机构，缩短了审批时限，方便了企业和群众办事。兴隆台区在全市率先实现了行政审批一站式服务。有效整合检验检测资源，区域性检验检测中心正在形成。

推进市和县区同权改革。将投资审批、重点行业许可、社会民生管理等416项市级权力全面赋予县区、经济区，同步推进财权与事权相匹配，进一步增强了县区、经济区发展活力。

深化国企改革。推进国资国企整合重组和经营性事业单位转企改制，在全省率先组建了“1+6+2”国有企业集团。农垦改革实现新突破，大洼被确定为国家深化农垦改革专项试点。完成农村集体土地承包经营权确权登记工作。

实施创新社会治理加强基层建设改革。推动政府职能、人员编制向镇街、村社区下沉，全面实施网格化管理，有力提升了基层治理和服务水平。

顺利实现大洼撤县设区，盘锦在新型城镇化、全域城市化建设上迈出了坚实一步。

（四）结构调整迈出新步伐

实施着力推进制造业优化升级专项行动。20个重大技改项目顺利实施，其中7个项目竣工投产。制定实施《中国制造2025盘锦行动纲要》，40个智能制造项目加快推进，陆海研究院、中蓝电子等6户企业成为全省首批智能制造及服务试点示范企业。生物质能、高效催化剂和无人机等新兴产业快速发展。

围绕推进服务业供给侧结构性改革，实施服务业发展质量提升三年行动。改造提升38条商业街区、5个主要集贸市场，餐饮住宿娱乐等行业向高品质方向发展。引进域外金融机构7家。新增新三板挂牌企业7户。设立20亿元的产业创业投资基金、10亿元的信贷风险补偿基金，融资担保体系不断完善。投放惠农贷10.5亿元。盘山县东北快递物流园开工建设，一批国内知名快递物流企业快速集聚。大洼电商模式被商务部评为十大经典案例在全国推广。金社裕农集团获得首批“全国公益性农产品市场零售企业”称号。

实施发展特色旅游业专项行动。全市旅游业总收入增长15.5%。成功举办第二届红海滩国际马拉松赛。红海滩国家风景廊道跻身全国体育旅游精品景区，“十一”黄金周接待游客32万人，位列全省各景区之首。冰凌穿越挑战赛获评全国体育旅游十佳精品线路。“百村万床”民宿工程取得积极进展，乡村旅游成为新的热点。开海节、插秧节、稻草艺术节等四季旅游活动精彩纷呈。全省旅游产业供给侧改革现场会在我市召开，盘锦成为首批创建国家全域旅游示范市。

实施加快转变农业发展方式、构建现代农业体系专项行动。发展认养农业21万亩。稻、蟹、泥鳅鱼等“一地多收”模式发展到7万亩，畜牧标准化规模养殖率达到85%。大米、河蟹品牌价值大幅提升。水稻生产综合机械化率达到92%。粮食产量118.8万吨，再创历史新高。现代农业示范区、农产品质量安全管理、产业融合等多项国家级试点示范创建工作全面推进，盘锦现代农业发展走在全省前列。

（五）创新创业开创新局面

实施促进大众创业万众创新专项行动。培育小巨人企业80家，发展楼宇经济65万平方米，3014户个体工商户转型升级为企业。在全省率先实行“五证合一、一照一码”商事登记制度，新增小微企业16万户，增长116%。

不断拓展创业空间。盘锦高新区“一区两园”获得省政府批准。发展各类双创载体49万平方米，在孵企业900户，新建成4个国家级众创空间。双台子区精细化工科技孵化基地入驻企业19家，化工助剂、生物医药产业初步形成。

不断完善创新体系。盘锦大学科技园、大连理工大学产业研究院创新能力明显增强。新建产业技术研究院6家、市级以上工程研究中心24家，组建创新联盟5个。吸引“千人计划”专家7名，引进博士以上高端人才50名。组织实施核心技术攻关20项。天意公司“旋转导向钻井系统”打破国外企业技术垄断，兴隆台区新技术、新经济蓬勃兴起。

（六）城乡建设和生态环境治理迈上新台阶

实施打造宜居乡村建设升级版专项行动。新建成165个示范村。农村燃气基本实现户户通。307个村实现24小时供水。

建成城乡一体的大环卫体系。启动城乡客运公交一体化改造工程。实施农村小型污水处理工程试点。推广农村燃气壁挂炉取暖。改造农垦危房11336户。盘锦获得住建部“中国人居环境范例奖”。石庙子村成为“中国美丽休闲乡村”，赵圈河镇入选全国首批特色小镇，宜居乡村建设带动形成了城乡一体化发展的巨大优势。

加快提升城市建设管理水平。中新线扩建、林丰路大桥竣工通车。实施棚户区改造专项行动，改造城市棚户区10227套。销售商品房252万平方米，房地产去库存成效明显。实施加强网格化管理智慧化建设专项行动，大数据中心、“智慧交通”等启动建设，盘锦获得工信部“宽带中国示范城市最佳实践奖”。大力推进“三城联创”，全国文明城市创建省级测评排名第一，国家卫生城市创建工作顺利通过初评，食品药品安全放心城市创建工作得到国家食安办高度肯定。

加大生态环境保护力度。开展国家蓝色海湾整治行动，实施了湿地修复工程。植树造林475万株。淘汰10吨以下燃煤小锅炉72台，淘汰注销黄标车和老旧车辆1.3万台，秸秆综合利用率达到86%，空气环境质量综合指数位居全省第三名。城镇污水处理率达到87%，水环境质量保持全省前列。投资2.8亿元的螃蟹沟(六零河)综合治理工程全面竣工，实现了水清、岸绿、景美。

（七）民生和社会事业有了新提升

在经济下行压力较大的情况下，持续加大民生投入力度，全市财政支出的75.5%用于民生领域。40件重点民生实事全部兑现。

城镇新增就业1.6万人，城镇登记失业率2.4%，低于控制目标0.6个百分点。企业退休人员基本养老金标准、城乡居民医疗保险政府补助标准分别提高到1575元和420元。城乡低保标准提高7%。居民大病保险实现城乡全覆盖。

狠抓精准扶贫，全市国标线以下贫困人口继续保持动态为零。辽河口经济区巩固提升工程取得显著成效，30项重点工程全面落实，群众生活进一步改善。为700户贫困残疾人家庭实施无障碍改造。新建扩建3个社会福利院，社区养老机构加快发展。

23所乡镇公办幼儿园全部开园。50所市直中小学校全面下放双台子区、兴隆台区管理。兴隆台区通过省级义务教育均衡发展工作验收。中考和普通高中招生改革不断深化，教育质量明显提高。贫困家庭学生和残疾学生资助实现全覆盖。盘锦成为国家技术技能人才双元培育改革试点市。

启动国家城市公立医院综合改革试点工作。建立医疗卫生服务四级联动体系，市中心医院建成“六大中心”，对全市基层医护人员实施集中培训，实现了对镇卫生院24小时远程会诊。计划生育政策得到全面落实。

积极创建国家公共文化服务体系示范区，文化惠民工程全面完成。文化产业加快发展，兴隆台区广厦艺术街跻身省级大学生创业示范基地。省第十三次运动会筹备工作顺利推进。全民健身活动蓬勃开展。

（八）法治政府和平安盘锦建设得到新加强

全面推进依法行政，自觉接受市人大及其常委会的法律监督、工作监督和市政协的民主监督，主动接受社会监督，人大代表建议、政协提案全部办复，满意率均达到100%。建立重大决策合法性审查制度，严格规范执法，加强政务公开，政府工作始终在法治的轨道上运行。盘锦被评为“全国法治宣传教育先进市”。

坚持常态化开展安全稳定调度评估，加大重点领域整治力度，我市未发生一起重特大安全生产事故、环境污染事故和食品药品安全事故，信访工作综合考核全省第一。严厉打击各类违法犯罪活动，侦破一批重大刑事案件，圆满完成20多项重大活动安保任务，公安执法规范化建设、社会治安立体化防控体系建设走在全省前列。

民族宗教、外事侨务、人民防空、妇女儿童、残疾人、气象、地震、统计、档案等工作取得新的进展。我市实现全国双拥模范城五连冠。

回顾过去的一年，我们真抓实干，锐意进取，推动盘锦发展向前迈出了坚实一步。

过去的一年，我们全面落实从严治党责任，深入开展“两学一做”学习教育，牢固树立“四个意识”，严守政治纪律和政治规矩，严肃认真地抓好中央巡视组“回头看”反馈意见整改落实工作，严格执行中央八项规定，加大廉政监察、审计监督力度，巩固提升了风清气正的良好政治生态。

过去的一年，我们牢牢抓住发展第一要务不动摇，坚持用国际化视野谋划盘锦发展，坚定不移抓改革促开放，集中精力抓产业上项目，持之以恒调结构转方式，积蓄了科学发展的新动能。我市多项工作在全省进行经验交流，国家多个部委来盘调研并给予充分肯定。

过去的一年，我们坚持站上国家平台推动盘锦建设，牢牢抓住中央振兴东北老工业基地重大政策“窗口期”，26个国家级改革创新试点示范政策落地见效，140个项目纳入国家重大项目建设库，对上争取资金突破100亿元，获批国家专项建设基金76.6亿元。盘锦的战略地位空前提升，为加快发展聚集了新优势。

过去的一年，我们坚持把工作思路转化为具体任务抓落实，坚持以项目化方式抓落实，坚持突出问题导向抓落实，把省委省政府和市委的决策部署、市七届人大四次会议确定的目标任务，细化分解为千余项具体任务，落实责任，明确时限，严格考核，形成了全市上下抓发展的强大合力。

过去的一年，我们始终保持时不我待的紧迫感和奋发有为的工作劲头，团结带领广大干部五加二、白加黑地奋战在一线，闯过了一个又一个难关，造就了一支想干事、能干事、干成事的干部队伍。事实证明，任何困难也挡不住盘锦前进的步伐！

过去一年的成绩来之不易。这是市委正确领导的结果，是市人大及其常委会、市政协监督支持的结果，是全市人民团结奋斗的结果。在此，我代表市政府，向全市人民，向各民主党派、工商联、人民团体和无党派人士，向驻盘部队、武警官兵、中省直机关企事业单位，向所有关心支持盘锦发展的各界朋友，表示衷心的感谢！

同时，我们也清醒地认识到，发展中还存在一些矛盾和问题。一是有效投资储备不足，项目建设力度需进一步加大；二是企业创新能力不强，人才队伍建设还不适应发展需要；三是软环境建设有待进一步加强，个别干部还存在不作为、不敢为等问题。对于这些问题，我们将在今后的工作中认真加以解决。

二、2017年工作总体要求

各位代表：市第七次党代会科学谋划了我市未来五年的宏伟蓝图，确立了加快建设国际化中等发达城市的奋斗目标，提出了用国际化视野谋划发展、站上国家平台推动建设的工作总要求，开启了建设滨海新盘锦的崭新篇章。

我们要建设的国际化中等发达城市，是一座市域经济强、全体百姓富、整体环境优、城乡人文美，保持中等发展规模、综合实力达到中等发达水平、具有盘锦特色的国际化城市。加快建设国际化中等发达城市，务必要坚持发展第一要务不动摇，把扩大有效投资作为长期坚持的重要抓手，聚精会神抓招商抓项目，用优质增量优化产业结构、提高供给质量，实现质量效益的同步提升；务必要坚持用国际化视野谋划盘锦发展，找准方向定位，拓展发展空间；务必要坚持站上国家平台推动盘锦建设，集聚国家优质资源要素，提升各项工作质量水平。

经过多年的不懈努力，盘锦奠定了建设国际化中等发达城市的现实基础，具备了在全省乃至全国城市发展中脱颖而出的竞争优势，站在了小城市大作为实现“盘锦梦”的历史关口，迈进了扎实稳步发展的重要机遇期、城市全面提质的历史跨越期、人民幸福的重要收获期。我们要团结带领全市人民，拼搏进取，真抓实干，为实现这个美好愿景而努力奋斗！

今年，是全面落实市第七次党代会精神、建设国际化中等发达城市的关键之年，也是实施“十三五”规划的重要一年。今年政府工作的总体要求是：全面贯彻党的十八大和十八届三中、四中、五中、六中全会以及中央经济工作会议精神，深入学习贯彻习近平总书记系列重要讲话精神，全面落实省第十二次党代会、省委经济工作会议和市第七次党代会、市委七届三次全会精神，适应把握引领经济发展新常态，持之以恒落实五大发展理念和“四个着力”，坚持用国际化视野谋划盘锦发展、站上国家平台推动盘锦建设，以推进供给侧结构性改革为主线，以提高发展质量和效益为中心，以扩大有效投资为重点，深入实施“四个驱动”，全面深化改革，持续扩大开放，推进城乡统筹发展，加快建设国际化中等发达城市，以优异成绩迎接党的十九大胜利召开。

着眼于建设国际化中等发达城市，结合“十三五”规划的实施，今年全市经济社会发展的主要预期目标是：地区生产总值增长7%；固定资产投资增长13%；规模以上工业增加值增长7.5%；一般公共预算收入增长10%；社会消费品零售总额增长9%；城镇常住居民人均可支配收入增长7%；农村常住居民人均可支配收入增长8.5%。

做好今年工作，我们要牢牢把握好以下四点：

第一，持之以恒落实“四个着力”。把“四个着力”作为推动发展的行动纲领，坚持落实“四个着力”与贯彻五大发展理念有机结合，与落实市第七次党代会精神和“十三五”规划有机结合，保持战略定力，开创盘锦科学发展的崭新局面。

第二，坚持不懈扩大对外开放。把扩大开放作为建设国际化中等发达城市的实现路径，主动顺应经济全球化发展趋势，积极融入“一带一路”、京津冀协同发展等国家开放战略，大规模引进来、大踏步走出去，以辽东湾新区为龙头推进全域全面开放，推动盘锦国际化建设在重点区域、优势产业、城市功能、社会人文等领域率先起步。

第三，毫不动摇全面深化改革。把改革作为建设国际化中等发达城市的关键之举，全面落实市委决策部署，统筹推进各领域改革，加大供给侧结构性改革、“放管服”改革等关键性基础性改革力度，破除束缚发展的体制机制障碍，更大激发市场活力和社会创造力。

第四，全力以赴狠抓工作落实。把抓落实作为政府工作的生命线，继续坚持业已形成的好的工作机制，坚持以项目化方式抓落实，坚持突出问题导向抓落实，坚持把工作思路转化为具体任务抓落实，确保党中央国务院、省委省政府和市委的决策部署不折不扣地落实到位。

三、今年主要工作任务

今年，要重点做好以下十个方面的工作：

（一）保持经济平稳增长

推动我市经济增长的关键在于扩大有效投资、支持企业发展。深入落实省委、省政府“项目年”的决策部署，坚持盘活存量、吸引增量、扩大总量，继续实施全面推进项目建设专项行动，狠抓 “六个一批”工程，亿元以上项目新开工90个、续建273个、竣工129个。

全力争取一批。认真贯彻落实国发62号、国办82号等文件精神，全力争取政策、项目和资金支持。争取将辽东湾新区纳入全国重点石化产业布局、我市成为国家产融合作试点市。

加快引进一批。建立专业化招商队伍，紧盯国内外重点区域，围绕优化产业结构、发展新兴产业，加大招商选资力度，力推一批在谈项目签约注册。

推进开工一批。落实项目推进责任，确保中国兵器集团精细化工及原料工程项目开工建设，推动台湾正新轮胎等16个重大项目落地开工。

抓紧竣工一批。加强项目分类管理和跟踪服务，促进续建项目加快建设、早日见效，支持北沥润滑油加氢、中储粮综合产业基地等9个重大项目竣工投产和运营。

改造提升一批。鼓励企业实施技术改造，重点抓好华锦石化改扩建、辽河石化润滑油加氢等13个重大技改项目和33个智能制造项目，提升企业竞争力。

合作共建一批。用好国家和省对口合作机制，加强与北京、江苏等发达地区在项目、技术、人才等领域的合作。推进诚通集团金融与旅游两大合作项目签约落地。建设盘锦北京亦庄产业园，打造北京产业转移主要承接地。

企业是发展的重要支撑。我们要把保障企业健康发展作为一项重要任务，全面落实“三去一降一补”系列政策措施。支持辽河油田稳产、改革和结构调整，鼓励华锦集团、辽河石化等大企业创新发展，引导中小企业消化库存、提质增效，帮助停产半停产企业尽快恢复生产。推动“银政企保”合作，千方百计帮助企业解决资金问题。推出降低企业成本、降低杠杆率等方面的政策措施，增强企业应对挑战的能力和信心。高度重视企业家队伍建设，弘扬“工匠精神”，培育和造就一大批优秀企业家，引领实体经济的大振兴、大发展！

（二）加快辽东湾新区建设

聚焦聚力推进新区建设，力争主要经济指标走在全省国家级开发区前列。

提升产业集聚水平。大力发展石化及精细化工、装备制造、临港加工、粮食物流等主导产业，发展现代服务业、旅游业等特色产业，着力打造国内外先进产业转移的承接地。重点抓好北燃公司炼化一体化、忠旺铝产业链生产基地、益海嘉里等20个重大项目，力争再引进项目30个以上，其中10亿元以上重大项目6个，实现固定资产投资的重大突破。

进一步创新体制机制。争取并用好国家和省级经济管理权限，推进行政审批制度改革，实施企业投资项目管理负面清单制度，开展零审批试点。复制落实自贸区改革措施，推动体制机制与国际接轨，建设东北一流的国际化营商环境。

继续实施打造辽东湾新区创业新城建设专项行动。按照国际化理念提升城市建设水平，全面实施25项基础设施工程，抓好文旅、商住等22项公共服务设施建设。提升曙光科技创业区和创业大厦等载体创业孵化能力，集聚人气商气。

（三）推进全域全面对外开放

按照国际先进标准推进港口与口岸建设。抓好盘锦港油品、粮食、液体化学品专业码头和通用泊位建设，加快实施10万吨级航道工程，吞吐能力达到6000万吨。完善港口集疏运体系，打通盘满欧、盘蒙欧国际陆路通道；开辟国际贸易线路，畅通盘海欧开放通道。提升一类口岸开放功能，申报国家粮食入境指定口岸，推动设立汽车整车进口口岸。加快口岸通关单一窗口建设，实现港口与口岸通关一体化。

打造在世界产业分工中占有一席之地的产业体系。瞄准产业开放的主攻方向和突破口，着力建设“一个核心特色”、“三大产业基地”，形成国际化中等发达城市的核心支撑。依托生态资源，发展全域旅游，创建国家级全域旅游示范区，建设世界生态休闲旅游目的地，让湿地生态成为盘锦对话世界的窗口、世界认识盘锦的最核心标志。依托重点石化产业园区，打造世界级石化及精细化工产业基地。依托港口和区位节点优势，打造区域性物流产业基地。依托高校和各类创业载体，打造具有一定影响力的区域性创新创业基地。

加快发展外向型经济。抓好保税物流中心运营，积极申建综合保税区。大力引进外资，实际利用外资3.5亿美元以上。加强出口基地建设，培育外贸综合服务企业，外贸出口5.6亿美元以上。支持有条件的企业走出去，推进国际产能和装备制造合作。

（四）加大重点领域改革攻坚力度

深入推进“放管服”改革。大力精简审批事项和前置要件，深入推进行政审批机构相对集中行政许可权改革，全面推行全程代办+容缺审批+企业承诺制度，抓好审批监管平台和审批事项标准化建设，进一步转变政府职能、提高行政效率。健全事中事后监管体系，实现“双随机一公开”监管全覆盖。推进网上行政服务中心建设，打造政务服务“一张网”。

深化投融资体制改革。促进国有资产集中统一监管全覆盖，建立“1+6+2”集团公司现代企业制度，实现集团公司市场化运营，打造新的经济增长点。充分发挥专项建设基金、产业投资基金带动作用。滚动推出一批PPP项目。做好驻盘央企“三供一业”分离移交工作。推进经营性事业单位转企，抓好行政类事业单位和公益类事业单位改革。

推进农垦企业改革。抓好大洼区农垦改革国家专项试点，释放国有农场土地资源优势，培育现代农业企业集团。落实国家农村承包土地“三权分置”办法，引导农户依法自愿有序流转土地经营权，发展多种形式的适度规模经营。

开展软环境建设年活动。实施软环境建设专项行动，坚决纠正不作为慢作为乱作为等行为，切实保护各类市场主体的合法权益。各级政府都要寓管理于服务，着重提高行业管理部门和窗口单位服务效能，构建既亲又清的政商关系，推动形成人人都是软环境、处处营造发展环境的浓厚氛围，让盘锦始终成为投资的热土、企业家的乐园！

（五）促进产业结构优化升级

积极推进三次产业供给侧结构性改革，坚持增量调结构，创新促升级，不断提高产业发展的质量和效益。

继续实施着力推进制造业优化升级专项行动。大力发展精细化工产业，延伸拉长石化产业链，提高原料加工度，重点抓好辽东湾新区精细化工产业园和双台子区精细化工科技孵化基地建设。做强装备制造业，促进能源装备规模化扩张、向高端化升级，培育铝合金制品、专用汽车、汽车零部件等产业链。加快发展无人机、电子信息、节能环保设备、应急救援、安全防护和新能源等新兴产业。

全面完成服务业发展质量提升三年行动计划，扩大现代服务业供给。积极发展金融业，加强银行、保险、证券等金融机构引进，做大做强市商业银行；完善融资担保体系和增信机制，引导金融机构更好地为企业服务；促进企业挂牌上市、债券发行等直接融资，金融业增加值增长7%。大力发展平台经济、快递物流等新兴服务业，重点抓好石化产品交易、粮食储运交易、跨境电子商务和智慧旅游四大平台建设，石化产品交易额达到150亿元以上。促进生活性服务业转型升级，商贸流通业增加值增长7.5%。

大力发展生态旅游业。丰富“红海滩+”旅游产品体系，抓好辽河口经济区旅游合作项目开发，确保红海滩国家风景廊道晋升为5A级景区、辽河国际艺术区晋升4A级景区。引进大型游乐公园等项目，打造中国旅游创意城市。完善吃住行游购娱一体化服务体系，突出抓好国际名品折扣购物中心项目引进，加快发展旅游特色纪念品、小商品等产业，建设区域购物中心，增强旅游消费拉动作用。依托休闲农业、民俗文化和景区优势，培育一批特色民宿村和旅游小镇，实现由观光向休闲度假的转变。加强旅游市场营销，提升稻草艺术节等品牌活动国际国内影响力，让四季旅游活动更加精彩，迎接正在兴起的大众旅游时代。全市旅游业总收入增长12%以上。

继续实施加快转变农业发展方式、构建现代农业体系专项行动，让盘锦现代农业建设始终走在全省前列。加大旱田区种植业结构调整力度，发展果蔬等高附加值经济作物。着力引进国内外知名企业参与大米、河蟹等资源整合和品牌创建，发展绿色农业、精品农业、高效农业、创汇农业。加快发展农产品加工及农产品物流产业，着力抓好京粮集团、汇福粮油等重大项目，建设北方最大的农产品加工和粮食仓储、物流产业基地。支持各镇因地制宜发展商贸流通、健康养老、轻工业等产业，增强镇域经济实力。落实粮食安全责任制，确保国家粮食安全。

加强全面质量管理。开展增品种提品质创品牌质量提升行动，实施以争创名牌产品、地标产品、品牌示范区为重点的品牌提升工程，不断扩大高质量产品供给。

（六）深入实施创新驱动发展战略

抓创新就是抓发展，谋创新就是谋未来。把创新作为引领国际化中等发达城市建设的第一动力，以不断提升壮大企业研发中心为重点，加快构建以创新为引领的发展新模式。

着力引进和建设科研院所。全力争取盘锦高新区晋升为国家级高新区。抓好产业技术研究院、企业研发中心和创新联盟引进，推进创新链与产业链有效对接，提高创新成果转化效率。引进产业技术研究院5家以上、创新团队5个以上。

加大企业研发投入力度。实施企业自主创新能力提升三年专项行动，充分发挥专项基金撬动作用，发展天使投资、创业投资、风险投资，引导企业增加研发投入。加强中蓝电子、智能装备等“园中园”建设。新建企业技术中心20家以上。新培育小巨人企业50家。

加快人才集聚。完善人才引进、激励和服务保障体系，加大政策宣传、落实力度，千方百计吸引和培育各类高层次创新创业型人才、急需紧缺型人才、具有国际化视野的企业管理人才和职业技能型人才。全年引进企业所需的各类人才2000人以上。

推动大众创业、万众创新。深化商事制度改革，推行个体工商户“两证整合”，进一步降低创新创业门槛。加强各类创业孵化载体建设，完善创新创业公共服务体系。大力推进“互联网+”，发展众创空间15家以上，促进新技术、新产品、新业态加快成长。

大力发展民营经济。民营经济是最具活力的增长力量，是就业的最大容纳器。出台支持民营经济发展的政策措施，积极营造良好发展环境，扩大民营经济发展规模。放宽民间资本准入行业和领域，促进民营企业公开公平公正参与市场竞争，扶持企业做大做强。引导民营企业和社会资本参与国资国企改革，支持民营企业与国有企业建立紧密的合作关系。充分发挥工商联及各类商会协会作用，形成推动民营经济大发展、快发展的合力，使民营经济成为盘锦经济发展的重要支撑。盘锦人杰地灵，富有创新创业的传统，我们一定能够站上时代发展的潮头！

（七）全力保障和改善民生

发展的根本目的是增进人民福祉，让百姓过上好日子。稳住就业基本盘、兜住社会保障底线，持续加大投入力度，着力解决好人民群众普遍关心的突出问题，继续办好民生实事。

坚持以创业带动就业，加强就业技能培训，城镇新增就业1.8万人，城镇登记失业率控制在3%以内。整合城乡居民基本医疗保险制度。积极推进城乡居民基本医疗保险支付方式改革。继续提高最低生活保障标准。加强社保基金扩面征缴和支出管理，防范企业养老金支付风险。抓好精准脱贫，保持国标线以下贫困人口动态为零。继续做好省内对口帮扶工作。

促进义务教育均衡发展，推动城乡教师有序流动。推行素质教育，狠抓师德师风建设，减轻学生课业负担，保障中小学生身心健康。推进学前教育普惠性发展。支持高等教育内涵式发展。开展职业教育技术技能人才双元培育试点，推动教育国际化。完善扶困助学体系，不让一名学生因贫失学。

挖掘辽河口文化，培育地域文化品牌。推动文化平台创新，搭建与世界文化交流的平台。抓好文化场馆、基层综合文化服务中心等公共文化设施建设，进一步完善农村文化广场功能，实施文化惠民工程，创建国家公共文化服务体系示范区。推进有线电视网络整合。大力发展文化产业，争取我市进入国家文化消费试点城市行列。

高标准办好“红马赛”、冰凌穿越挑战赛和亚洲国际象棋赛，推动体育国际化。全力筹备省第十三届运动会。广泛开展全民健身活动，改造提升全民健身中心。

推进健康盘锦建设。制定“健康盘锦2030”规划，全方位全周期保障群众健康。推进区域性检验检测中心建设。深化城市公立医院改革，增强基层卫生服务能力。全面实施分级诊疗，为农村居民开展远程会诊。为65岁以上老年人开展医疗卫生签约服务。为低收入群体免费实施1000例白内障复明手术。为部分农村妇女、城乡低保家庭妇女免费开展“两癌”筛查。提升中医药服务能力。落实计划生育政策，强化生育关怀。推进医养结合养老服务，抓好公益性养老，发展日间照料和居家养老。

加强财税管理。坚持依法依规征管，多措并举应收尽收。调整优化支出结构，重点保民生、保运转。

（八）加强城乡建设和生态环境保护

坚持国际化引领、城乡统筹协调、全域生态建设，加强城市总体设计。推进多规合一，统筹专项规划，强化刚性约束，维护规划的权威性和严肃性。

推进城市建设与管理。竣工沈盘铁路，推进阜盘铁路工程，建设朝盘铁路，启动疏港铁路客运化改造。推进京哈高速扩容、高升至西安高速公路项目，力争绕城路(东外环)建成通车。实施城市交通管理专项行动，构建现代化智能化大交通体系，打造符合国际化中等发达城市标准的特色交通。支持双台子区等老城区改造。加快建设地下综合管廊工程。实施城市棚户区改造7770户。加大房地产去库存力度，商品房销售面积增长10%。加强建设工程质量管理，建筑业总产值增长10%。实施加强城市精细化管理专项行动，提升城市智能化精细化网格化管理水平。推进“多城联创”，晋升全国文明城市、国家卫生城市，争创国家食品药品安全放心城市和国家园林城市。

继续实施打造宜居乡村建设升级版专项行动。巩固扩大宜居乡村建设成果，推进城乡基本公共服务均等化，实现示范村全覆盖。提升农村饮水安全水平，完成70个村水源替代工程。推进城乡客运公交一体化，完成135台客运车辆公交化改造任务，建设52个农村候车厅。加快推进农村污水治理工程，完成100个村小型污水处理设施建设。农村燃气覆盖率达到100%。推进冬季清洁取暖，继续推广使用农村燃气壁挂炉。加快建立垃圾分类处理系统。提升畜禽养殖废弃物处理和资源化利用水平。落实宜居乡村建设长效管理机制，进一步把宜居乡村建设优势转化为产业发展优势。

实施加强生态建设专项行动。开展国家生态文明示范市创建工作，让绿色成为盘锦最厚重的底色，让生态环境成为城市的金字招牌。科学划定生态红线。认真实施《盘锦市湿地保护条例》，加大湿地保护与修复力度，创建国家级海洋生态文明示范区。开展国际滨海湿地保护利用论坛等国际性活动。实施全域绿化工程，城市要提升园林园艺水平，打造花园式工厂、花园式机关和花园式学校；农村要见缝插绿，促进乡村园林化、庭院绿化花果化。全年新建和改造提升城市公园6座，改扩建绿地面积266公顷。实施蓝天工程，拆除老旧低效燃煤锅炉82台，淘汰黄标车及老旧车辆6171辆；强化扬尘整治和秸秆禁烧，开展石化企业挥发性有机污染物治理，推动空气质量持续好转。实施碧水工程，加强水源地保护，抓好污水处理厂改造提标；推进河流水系连通工程，打造河网景观。实施沃土工程，强化土壤污染治理与修复。落实最严格的环境保护制度，构建源头控制、过程监管、末端治理同步发力的环境管理链条，促进绿色低碳循环发展。

（九）推进法治建设和社会治理创新

坚持依法行政。把政府工作全部纳入法治轨道，依法接受市人大的法律监督和工作监督，认真落实市人大及其常委会的各项决议决定，主动接受市政协的民主监督。推进立法工作，严格依法执法，加强执法监督和政务公开，让权力在阳光下运行。全面实施“七五”普法规划，提高全民法治意识，建设法治盘锦、信用盘锦。

创新社会治理方式。抓好智慧城市大数据应用平台和一站式公共服务平台建设。继续实施创新社会治理加强基层建设专项行动，建立智慧城市运行管理系统，实现四级网格管理平台互联互通，提升基层社会治理和服务能力。推进社会管理、公共服务等领域现代化建设。

推进平安盘锦建设。加强安全稳定常态化调度评估，坚决防范重特大安全事故发生。完善矛盾纠纷多元化解机制，维护群众合法权益。深入推进公安执法规范化建设。完善立体化信息化社会治安防控体系，开展常态化社会治安打击整治专项行动。蝉联“长安杯”。

支持工会、共青团、妇联等群团组织以及各类社会组织参与社会治理。强化国防教育和后备力量建设，积极支持军队改革，深入开展双拥共建工作。继续做好民族宗教、外事侨务、气象地震、防灾减灾、邮政通信、妇女儿童、老龄慈善、红十字会、质监物价、统计档案、地方志等工作。

（十）加强政府自身建设

面对艰巨任务，肩负重大使命，我们要按照建设法治政府、创新政府、廉洁政府、服务型政府的要求，切实加强政府自身建设。

我们要对标看齐，从严治政。切实增强“四个意识”，自觉向以习近平同志为核心的党中央看齐，坚决维护党中央的权威和集中统一领导。全面落实市政府党组从严治党责任，严肃党内政治生活，完善党内监督制度，从严抓好干部管理，不断提高政府的执行力。

我们要主动担当，真抓实干。始终保持昂扬向上、奋发有为的创业激情和工作劲头，时刻把“四个着力”抓在手上，把人民福祉挂在心上，扭住经济建设中心绝不动摇。建立容错纠错激励机制，激发责任意识，倡导担当精神，弘扬实干作风，切实为干事者撑腰、为负责者负责、为担当者担当，让改革创新、干事创业、务实奉献成为全市上下的自觉行动和共同追求。

我们要廉洁从政，干净干事。认真落实党风廉政建设各项规定，不断完善防腐治腐的制度体系，强化行政监察和审计监督，持之以恒加强和改进作风建设，推动党风廉政建设和反腐败斗争取得新成效，让各级干部正确用权、谨慎用权、干净用权。

各位代表，新的一年开启新的希望，新的征程承载新的梦想。让我们更加紧密地团结在以习近平同志为核心的党中央周围，在市委的坚强领导下，真抓实干，开拓创新，奋力谱写建设国际化中等发达城市的新篇章，以实际行动和优异成绩迎接党的十九大胜利召开！
